# MICAL2 contributes to gastric cancer cell migration via Cdc42-dependent activation of E-cadherin/β-catenin signaling pathway

**DOI:** 10.1186/s12964-022-00952-x

**Published:** 2022-09-05

**Authors:** Qianwen Wang, Chenxiang Qi, Pengxiang Min, Yueyuan Wang, Fengwen Ye, Tianxiang Xia, Yujie Zhang, Jun Du

**Affiliations:** 1 Department of Physiology, Nanjing Medical University, 101 Longmian Avenue, Jiangning District, Nanjing, 211166 Jiangsu China; 2 Key Laboratory of Cardiovascular and Cerebrovascular Medicine, School of Pharmacy, Nanjing Medical University, Nanjing, 211166 Jiangsu China; 3 Experimental Teaching Center of Basic Medicine, Nanjing Medical University, Nanjing, 211166 Jiangsu China

**Keywords:** MICAL2, Gastric cancer, Migration, E-cadherin/β-catenin complex

## Abstract

**Background:**

Gastric cancer is a common and lethal human malignancy worldwide and cancer cell metastasis is the leading cause of cancer-related mortality. MICAL2, a flavoprotein monooxygenase, is an important regulator of epithelial-to-mesenchymal transition. The aim of this study was to explore the effects of MICAL2 on gastric cancer cell migration and determine the underlying molecular mechanisms.

**Methods:**

Cell migration was examined by wound healing and transwell assays. Changes in E-cadherin/β-catenin signaling were determined by qPCR and analysis of cytoplasmic and nuclear protein fractions. E-cadherin/β-catenin binding was determined by co-immunoprecipitation assays. Cdc42 activity was examined by pulldown assay.

**Results:**

MICAL2 was highly expressed in gastric cancer tissues. The knockdown of MICAL2 significantly attenuated migratory ability and β-catenin nuclear translocation in gastric cancer cells while LiCl treatment, an inhibitor of GSK3β, reversed these MICAL2 knockdown-induced effects. Meanwhile, E-cadherin expression was markedly enhanced in MICAL2-depleted cells. MICAL2 knockdown led to a significant attenuation of E-cadherin ubiquitination and degradation in a Cdc42-dependent manner, then enhanced E-cadherin/β-catenin binding, and reduced β-catenin nuclear translocation.

**Conclusions:**

Together, our results indicated that MICAL2 promotes E-cadherin ubiquitination and degradation, leading to enhanced β-catenin signaling via the disruption of the E-cadherin/β-catenin complex and, consequently, the promotion of gastric cell migration.

**Video Abstract**

**Supplementary Information:**

The online version contains supplementary material available at 10.1186/s12964-022-00952-x.

## Introduction

Gastric cancer is among the most commonly diagnosed malignancies worldwide. There is a correlation between the severity of tumor metastasis and mortality in gastric cancer patients [1]. The migration of cancer cells into surrounding tissue represents the first step in tumor metastasis. Cytoskeleton dynamics, regulated by various actin-binding proteins, is considered to play an important role in cell migration [2, 3]. Accordingly, understanding the mechanisms underlying how actin-binding proteins control gastric cancer cell migration may lead to the identification of targets for attenuating gastric cancer cell metastasis.

The molecules interacting with CasL (MICALs) comprise a family of conserved flavoprotein oxidoreductases known to drive F-actin oxidization and participate in multiple cellular functions related to cytoskeleton dynamics, including vesicle transportation, mitosis, and the development of hippocampal mossy fiber connections [4–7]. In humans, the MICAL family of proteins consists of MICAL1, MICAL2, MICAL3, MICAL-like protein 1 (MICAL-L1), and MICAL-L2. Although MICAL1 is auto-inhibited by its C-terminal coiled-coil domain, MICAL2 lacks this region and remains constitutively active [8]. MICAL2 is highly expressed in several types of aggressive, poorly differentiated human epithelial cancers, as well as in cancer-associated neo-angiogenic capillary endothelia [9–12]. In addition, the silencing of MICAL2 has been reported to significantly inhibit cell growth and metastasis. For instance, in pulmonary arterial smooth muscle cells, miR-205-5p was found to attenuate MICAL2 expression, thereby blocking MICAL2-mediated promotion of cell proliferation [13]. In human kidney cancer cells, MICAL2 silencing induced epithelial-to-mesenchymal transition (EMT), reduced cell viability, and led to a loss of motility and invasive potential [14]. Additionally, MICAL2 overexpression can induce ROS production and activation of the SRF/MRTF-A and semaphorin/plexin pathways. Moreover, MICAL2 was recently identified as being a tumor-promoting factor able to accelerate tumor progression [15, 16]. However, to the best of our knowledge, the effects of MICAL2 on gastric cancer cell migration and the putative underlying molecular mechanisms remain largely unknown.

β-catenin is a dual function protein, both coordinating intercellular adhesion at adherens junctions and regulating the transcription of target genes. β-catenin forms a complex with E-cadherin, which, in turn, can function as an anchoring junction and act to stabilize cell adhesion [17]. When a Wnt ligand binds to its corresponding receptor, it can inhibit the cytoplasmic β-catenin destruction complex, allowing β-catenin to accumulate and translocate into the nucleus, where it mediates the transcription of Wnt target genes [18]. Increased β-catenin nuclear localization can influence a variety of cellular processes, including the breaking of cell-to-cell adhesion and increasing the cell migration potential [19]. Besides Wnt ligands, β-catenin nuclear accumulation is also regulated by several other molecules [20, 21]. A recent study revealed that MICAL-L2 contributes to Wnt/β-catenin signaling activation in ovarian cancer cells and that the silencing of MICAL-L2 abrogates the nuclear translocation of β-catenin and induces EMT [22]. Although MICAL2 differs from MICAL-L2 in that it lacks a CC domain and possesses a FAD domain, the two proteins share high sequence similarity [23]. Thus, we hypothesized that MICAL2 might also promote β-catenin nuclear translocation. To the best of our knowledge, no correlation between MICAL2 and β-catenin in gastric cancer cell migration has been identified to date. In the present study, we investigated the relationship between MICAL2 and β-catenin intracellular distribution in gastric cancer cells and explored the underlying mechanism, aiming to determine their importance as predictors of gastric cancer cell metastasis.

## Materials and methods

### Ethics statement

All immunohistochemical assays involving human tumor specimens were conducted according to the institutional guidelines of Jiangsu Province.

### Cell culture

The human gastric cancer cell lines BGC-823, MGC-803, and SGC-7901 and the normal gastric epithelial cell line GES-1 were purchased from the Cell Biology Institute of the Chinese Academy of Science (Shanghai, China). All cells were grown in Dulbecco’s modified Eagle’s medium (Hyclone, Thermo Fisher Scientific, Waltham, MA, USA) supplemented with 10% fetal bovine serum (Gibco, Carlsbad, CA, USA) and antibiotics (100 U/mL penicillin and 100 μg/mL streptomycin) (Invitrogen, Carlsbad, CA, USA). Cells were maintained in a humidified incubator at 37 °C with 5% CO_2_. Cells were grown on coverslips for immunofluorescence staining and in six-well plates (Costar, Corning, NY, USA) for RNA isolation and protein extraction.

### Plasmids and siRNAs

The empty vector control pcDNA-3.1-HA-C and full-length human MICAL2 cDNA were purchased from YouBio (Changsha, China). The pEGFP-N1 vector containing the full-length Cdc42-Q61L (CA) insert was kept in this laboratory. The siRNAs (control siRNA or siRNA targeting MICAL2 [siMICAL2]) used in this study were synthesized and purified by GenePharma (Shanghai, China). The sequences of the siRNAs targeting MICAL2 were as follows: siMICAL2 #1, 5′-GAGAACGUGAACCAAGACATT-3′; siMICAL2 #2, 5′-GCAUAGAUCUUGAGAACAUTT-3′; siMICAL2 #3, 5′-GCAGCGACACGUGUUACUUTT-3′. Transfection (plasmids or siRNA) was performed using Lipofectamine 2000 following the manufacturer’s instructions. After 24 h of transfection, the cells were cultured in starvation medium overnight and then treated with cycloheximide (CHX) (Sigma-Aldrich, Saint Louis, MI, USA), LiCl (Sigma-Aldrich), MG-132 (Selleck Chemicals, Houston, TX, USA), or chloroquine diphosphate (Chlq) (MedChemExpress, Monmouth, IL, USA) at the indicated time points.

### Wound healing assay

For the wound healing assay, cells were seeded in six-well plates until confluence. A wound was then made in the cell monolayer by scratching with a 10-µL pipette tip. After rinsing with PBS, the cells were treated with the indicated stimulator and allowed to migrate for 24 h. Images of wound areas were captured using an inverted phase-contrast microscope (Carl Zeiss Meditec, Jena, Germany) at 0 h and 24 h.

### Transwell assay

The transwell assay was performed using a 24-well cell culture insert with 8-μm pores. A total of 1 × 10^4^ cells were seeded in the upper chamber in serum-free medium while medium containing 10% FBS was added to the lower chamber. After incubation at 37 °C for 24 h, the cells on the upper side of the membrane (non-migrated cells) were removed while those that had migrated to the underside of the membrane were fixed in 4% paraformaldehyde and stained with 0.1% crystal violet. The average number of cells was determined and scored under a fluorescence microscope (Carl Zeiss Meditec).

### Western blotting

Cell lysates were prepared using RIPA lysis buffer (Beyotime, Shanghai, China). Cytoplasmic and nuclear protein fractions were obtained using a nuclear protein extraction kit from Beyotime following the manufacturer’s instructions. Protein concentrations were assessed using a BCA protein assay kit (Thermo Fisher Scientific). Equal amounts of cellular protein were separated by SDS–PAGE, transferred to a pure nitrocellulose membrane, blocked with 5% skimmed milk, and then incubated with primary antibodies targeting MICAL2 (Affinity, Cincinnati, OH, USA), GAPDH (Bioworld, Nanjing, China), E-cadherin (Proteintech, Wuhan, China), N-cadherin (BD Biosciences, New Jersey, USA), β-catenin, vimentin, Cdc42, and HA-tag (all from Cell Signaling Technology, Danvers, MA, USA) overnight at 4 °C. After washing and incubation with secondary antibody (HRP-conjugated normal rabbit IgG; Jackson, Lancaster, PA, USA), the bands were visualized using enhanced chemiluminescence reagent (FuDeBio, HangZhou, China) and analyzed using Quantity One software (Bio-Rad, Hercules, CA, USA).

### Real-time quantitative PCR

Total cellular RNA was isolated using Trizol reagent (Invitrogen) and reverse-transcribed with HiScript Q RT SuperMix for qPCR (Vazyme, Nanjing, China). The sequences of the primers used were GAPDH: 5′-TCGGATCAACGGATTTGGT-3′ (sense) and 5′-TTCCCGTTCTCAGCCTTGAC-3′ (antisense); MICAL2: 5′-GGGGATTTCCCGCAGAATAAAC-3′ (sense) and 5′-GGCTGGGATGAAAATGGAACC-3′ (antisense); vimentin: 5′-AGTCCACTGAGTACCGGAGAC-3′ (sense) and 5′-CATTTCACGCATCTGGCGTTC-3′ (antisense); E-cadherin: 5′-ATTTTTCCCTCGACACCCGAT-3′ (sense) and 5′-TCCCAGGCGTAGACCAAGA-3′ (antisense); N-cadherin: 5′-AGCCAACCTTAACTGAGGAGT-3′ (sense) and 5′-GGCAAGTTGATTGGAGGGATG-3′ (antisense). Real-time PCR was carried out using AceQ qPCR SYBR Green Master Mix (High ROX Premixed) (Vazyme) in an ABI StepOne Real-Time PCR System (Applied Biosystems, Foster City, CA, USA). Relative mRNA expression levels were calculated using the 2^−ΔΔCt^ method and StepOne Software v2.1 (Applied Biosystems).

### Immunofluorescence microscopy

Cells were placed on a glass coverslip, fixed in 4% paraformaldehyde for 20 min, treated with 0.2% Triton X-100 for 5 min, blocked with 1% BSA for 1.5 h, and then incubated with primary antibodies against β-catenin, E-cadherin, EEA1, Rab7 (all Cell Signaling Technology), and LAPM1 (Proteintech) overnight. After washing three times with PBS, the cells were incubated with Alexa-conjugated species-matched secondary antibody or TRITC-conjugated secondary antibody for 1 h. The nuclei were counterstained with DAPI (Beyotime). Images were captured using an Olympus BX51 microscope fitted with an Olympus DP70 digital camera (Olympus, Tokyo, Japan).

### Pulldown assay

Rho GTPase pulldown assays were performed as previously described [24]. Active Cdc42 was pulled down using PAK-CRIB beads. Briefly, cells were lysed, the lysates were centrifuged, the supernatants were transferred to new tubes containing beads pre-coupled with PAK-CRIB, and incubated under rotation at 4 °C for 30 min. The beads were subsequently washed and the proteins bound on the beads were separated by SDS–PAGE. The quantity of active Cdc42 was determined by western blot using the corresponding antibodies.

### Co-immunoprecipitation (Co-IP) assay

Co-IP was conducted as previously described [25]. In brief, a protein mixture was prepared and incubated with antibody-bound affinity beads (Beyotime). Once unbound proteins had been cleared using multiple washing steps, interacting proteins were detected via SDS–PAGE and western blot.

### Clinicopathological analysis of MICAL2

The Oncomine and UALCAN databases were used to analyze the association between the mRNA or protein expression of MICAL2 and clinical parameters as well as the prognostic value of mRNA expression of MICAL2 in gastric cancer tissue. Patients with gastric cancer were divided into high and low MICAL2 expression groups based on the median values of MICAL2 mRNA expression.

### Statistical analysis

Data were analyzed using SPSS version 19.0 (SPSS, Chicago, IL, USA) and are reported as means ± standard error of the mean. The Student’s *t*-test was used for comparisons between two groups. One-way ANOVA was utilized for comparisons among three or more groups. A *P*-value < 0.05 was considered significant. All experiments were repeated independently at least three times.

## Results

### MICAL2 was overexpressed in human gastric cancer samples

**Fig. 1:**
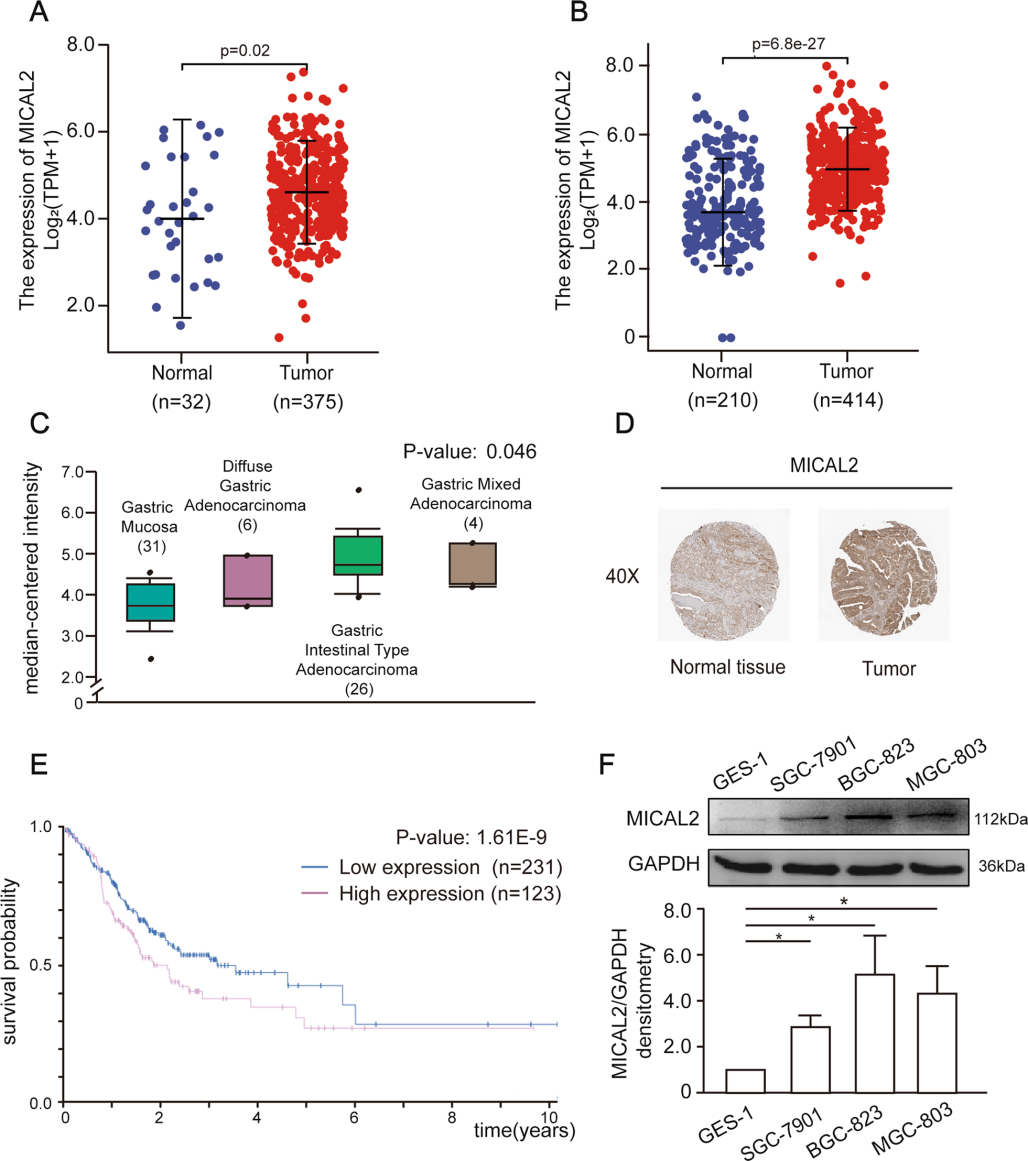
MICAL2 expression in gastric cancer tissues and cells. **A** Differences in MICAL2 expression in The Cancer Genome Atlas Stomach Adenocarcinoma (TCGA-STAD) tissues and adjacent normal tissues. **B** Differences in MICAL2 expression between TCGA-STAD cohort and normal samples in the Genotype-Tissue Expression (GTEx) database combined with adjacent TCGA-STAD tissue samples. **C** The MICAL2 mRNA level was assessed in normal gastric mucosa (*n* = 31), diffuse gastric adenocarcinoma (*n* = 6), gastric intestinal-type adenocarcinoma (*n* = 26), and gastric mixed adenocarcinoma (*n* = 4). Data from Gluck et al. **D** Representative images of MICAL2 staining in gastric cancer tissues and their normal controls from the Human Protein Atlas (HPA). **E** Kaplan–Meier analysis of overall survival (OS) for patients with low or high levels of MICAL2 expression. **F** MICAL2 protein levels in GES-1, SGC-7901, BGC-823, and MGC-803 cells were determined by western blotting assays. Data in (**F**) are presented as means ± SEM of three determinations. *P < 0.05

To investigate whether MICAL2 was associated with the pathogenesis and progression of gastric cancer in humans, we first assessed whether any correlation existed between the MICAL2 expression level and clinical parameters in gastric cancer. For this, we firsts analyzed the mRNA levels of MICAL2 in The Cancer Genome Atlas Stomach Adenocarcinoma (TCGA-STAD) dataset, and found that MICAL2 expression was significantly higher in tumor tissues than in normal tissues (Fig. 1A). We further compared MICAL2 expression between gastric cancer tissue from the TCGA-STAD cohort and normal samples in the Genotype-Tissue Expression (GTEx) database combined with adjacent TCGA-STAD tissue samples (controls), and obtained similar results (Fig. 1B). Data from Oncomine showed that MICAL2 expression was significantly higher in diffuse gastric adenocarcinoma, gastric intestinal-type adenocarcinoma, and gastric mixed adenocarcinoma than in normal gastric mucosa (Fig. 1C). Using the gastric cancer dataset from the Human Protein Atlas (HPA) (www.proteinatlas.org), we confirmed that MICAL2 was strongly expressed in the cytoplasm/membrane of gastric cancer samples (Fig. 1D). Kaplan–Meier survival curve analysis indicated that patients with high levels of MICAL2 expression had markedly shorter survival time compared with those with low MICAL2 expression levels (Fig. 1E). Next, we analyzed the expression of MICAL2 in three gastric cancer cell lines (BGC-823, MGC-803, and SGC-7901) and a non-malignant gastric epithelial cell line (GES-1; control) by immunoblotting. As expected, MICAL2 expression was higher in gastric cancer cells than in control cells (Fig. 1F). Overall, these data indicated that MICAL2 expression is upregulated in gastric cancer at both the mRNA and protein levels, and this increased expression might be associated with poor prognosis among gastric cancer patients.

### The effect of MICAL2 on gastric cancer cell migration and EMT markers

**Fig. 2:**
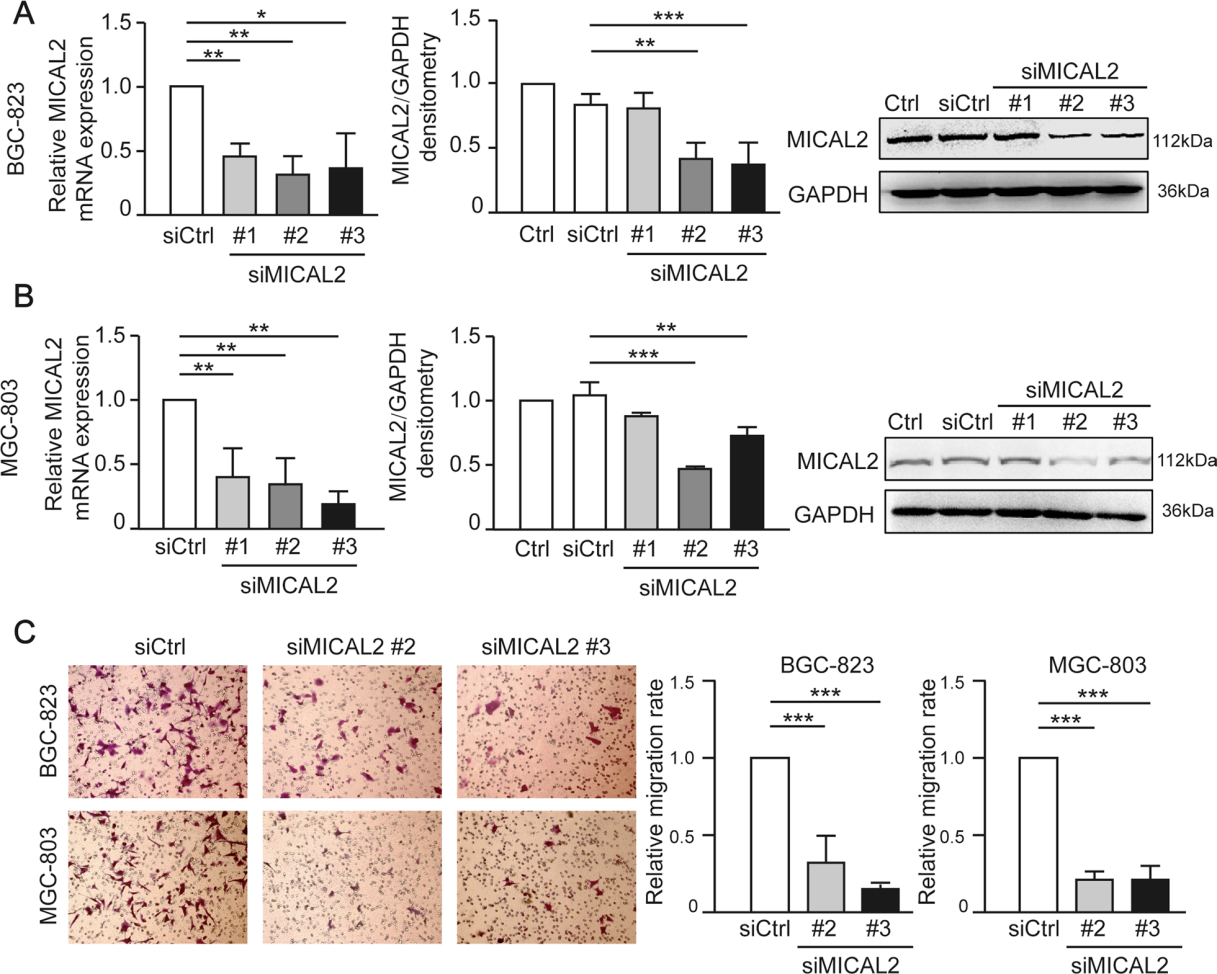
MICAL2 regulates the migration of human gastric cancer cells. **A**, **B** BGC-823 and MGC-803 cells were transfected with control siRNA or siRNA targeting MICAL2 (siMICAL2). Total mRNA and protein extracts from these cells were analyzed for MICAL2 expression. Western blot bands corresponding to MICAL2 were quantified and normalized against GAPDH (*n* = 3 per group). **C** Representative images of transwell assays in BGC-823 and MGC-803 cells transfected with control siRNA or siMICAL2 and quantification of the cell migration rate (*n* = 5 per group). *P < 0.05, **P < 0.01, ***P < 0.001

To confirm the role of MICAL2 in the regulation of cell migration, we performed MICAL2 loss-of-function assays in gastric cancer cells. First, we silenced MICAL2 expression in BGC-823 and MGC-803 cells using siMICAL2. The knockdown efficiency was determined by qPCR and western blotting. As shown in Fig. 2A and B, transfection with siMICAL2 #2 and #3 led to a significant reduction in MICAL2 expression in both cell lines when compared with that in control cells. Next, the effect of MICAL2 depletion on gastric cancer cell migration was evaluated by transwell assay. The results showed that the silencing of MICAL2 effectively impaired the migratory potential of both BGC-823 and MGC-803 cells (Fig. 2C).

**Fig. 3:**
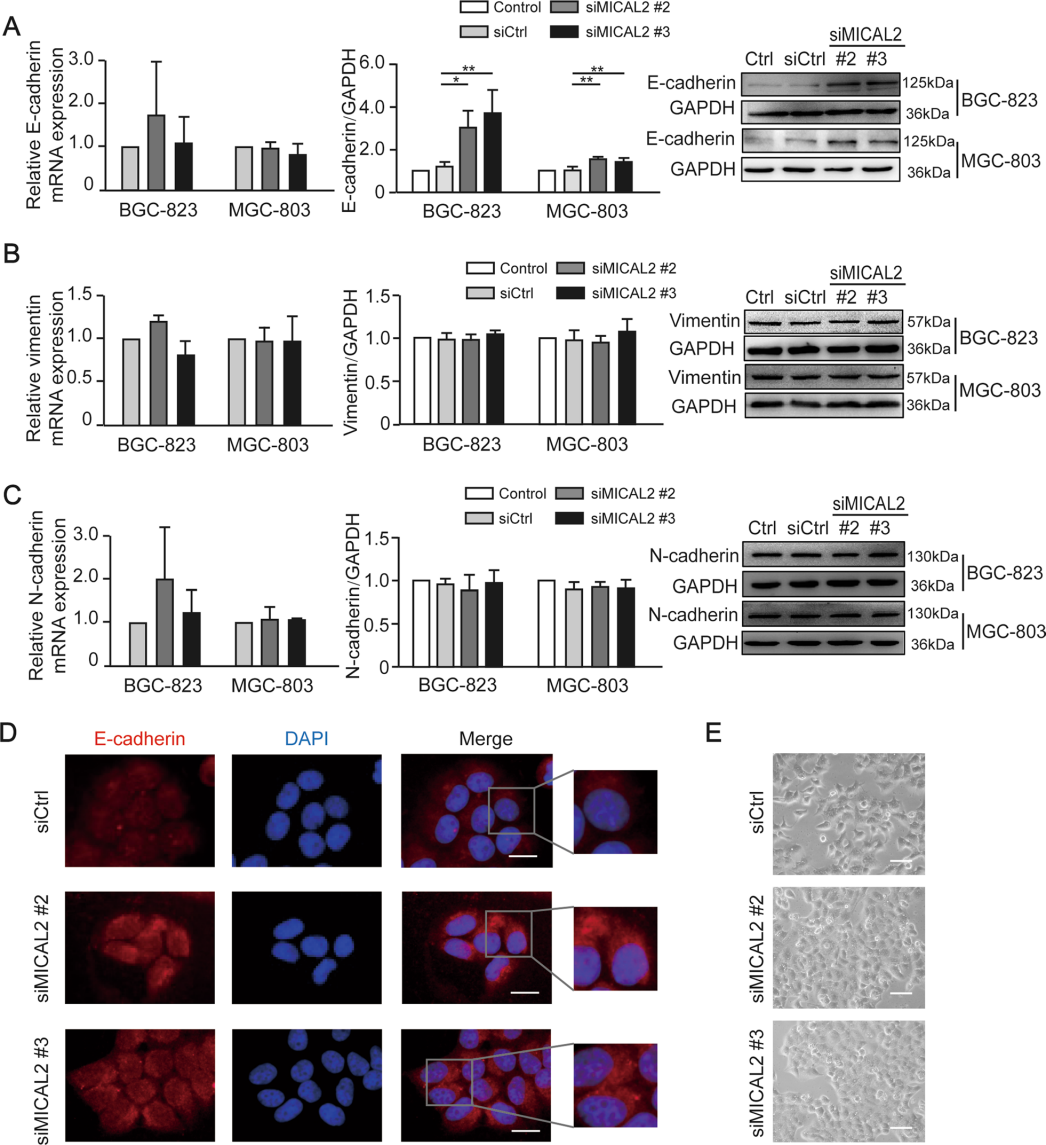
The effect of MICAL2 knockdown on the expression of epithelial-to-mesenchymal (EMT)-related marker proteins. **A**–**C** BGC-823 and MGC-803 cells were transfected with control siRNA or siRNA targeting MICAL2 (siMICAL2) following which the levels of E-cadherin (**A**), vimentin (**B**), and N-cadherin (**C**) were detected by qPCR and western blotting. **D** Representative immunofluorescence micrographs of BGC-823 and MGC-803 cells stained for E-cadherin. Scale bar, 10 μm. **E** The morphology of BGC-823 cells transfected with control siRNA or siMICAL2 was visualized using an inverted microscope. Scale bar, 20 μm. *P < 0.05, **P < 0.01

**Fig. 4:**
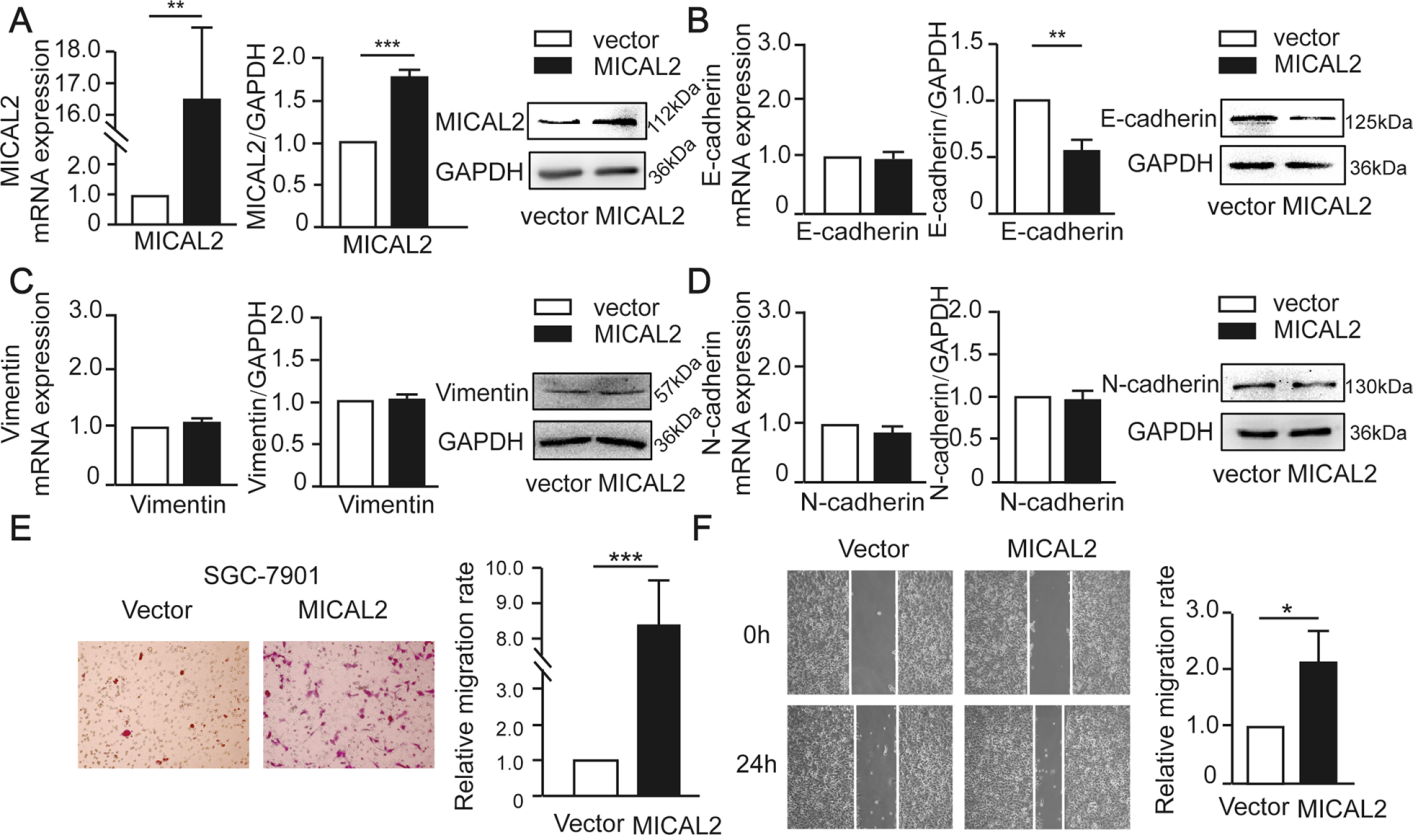
The effect of MICAL2 overexpression on epithelial-to-mesenchymal (EMT)-related marker proteins. **A**–**D** SGC-7901 cells were transfected with empty vector or a MICAL2 expression plasmid, after which the levels of MICAL2 (**A**), E-cadherin (**B**), vimentin (**C**), and N-cadherin (**D**) were measured by qPCR and western blotting. **E** Representative image of transwell assays in SGC-7901 cells transfected with empty vector or a MICAL2 expression plasmid. Cells on the lower surface of the membrane were quantified in three randomly selected fields and are shown in the panels on the right. **F** Representative image of wound healing assays in MICAL2-overexpressing SGC-7901 cells (*n* = 5 per group). Data are presented as means ± SD of the wound area relative to the control group. *P < 0.05, **P < 0.01, ***P < 0.001

We next tested whether MICAL2 modulates the expression of EMT markers in gastric cancer cells. BGC-823 and MGC-803 cells were transfected with siMICAL2 #2 and #3, following which vimentin, N-cadherin, and E-cadherin mRNA and protein levels were evaluated by qPCR and western blotting, respectively. Whereas BGC-823 and MGC-803 cells underwent markable reduction of MICAL2, no significant increase in the abundance of E-cadherin mRNA was observed (Fig. 3A). These findings suggested that the MICAL2-mediated modulation of E-cadherin expression was likely to be transcription-independent and may instead involve the suppression of E-cadherin degradation. No differences in vimentin and N-cadherin expression levels were detected (Fig. 3B, C). Immunofluorescence assays also revealed that the cytoplasmic/membrane content of E-cadherin was increased in MICAL2-silenced MGC-803 cells when compared with that in control cells (Fig. 3D). As expected, we noticed that MICAL2-depleted MGC-803 cells acquired some epithelial morphology (Fig. 3E). In contrast, overexpressing MICAL2 in SGC-7901 cells (Fig. 4A) led to decreased E-cadherin protein expression relative to that in control cells; however, the E-cadherin mRNA level was not affected (Fig. 4B). No significant differences in the mRNA and protein contents of vimentin and N-cadherin were detected in MICAL2-overexpressing SGC-7901 cells (Fig. 4C, D). Meanwhile, compared with the controls, MICAL2 overexpression resulted in a notable increase in the migration rate of SGC-7901 cells (Fig. 4E, F). These results suggested that MICAL2 positively regulates gastric cancer cell migration, particularly by depressing E-cadherin protein.

### The effect of MICAL2 on E-cadherin degradation

**Fig. 5:**
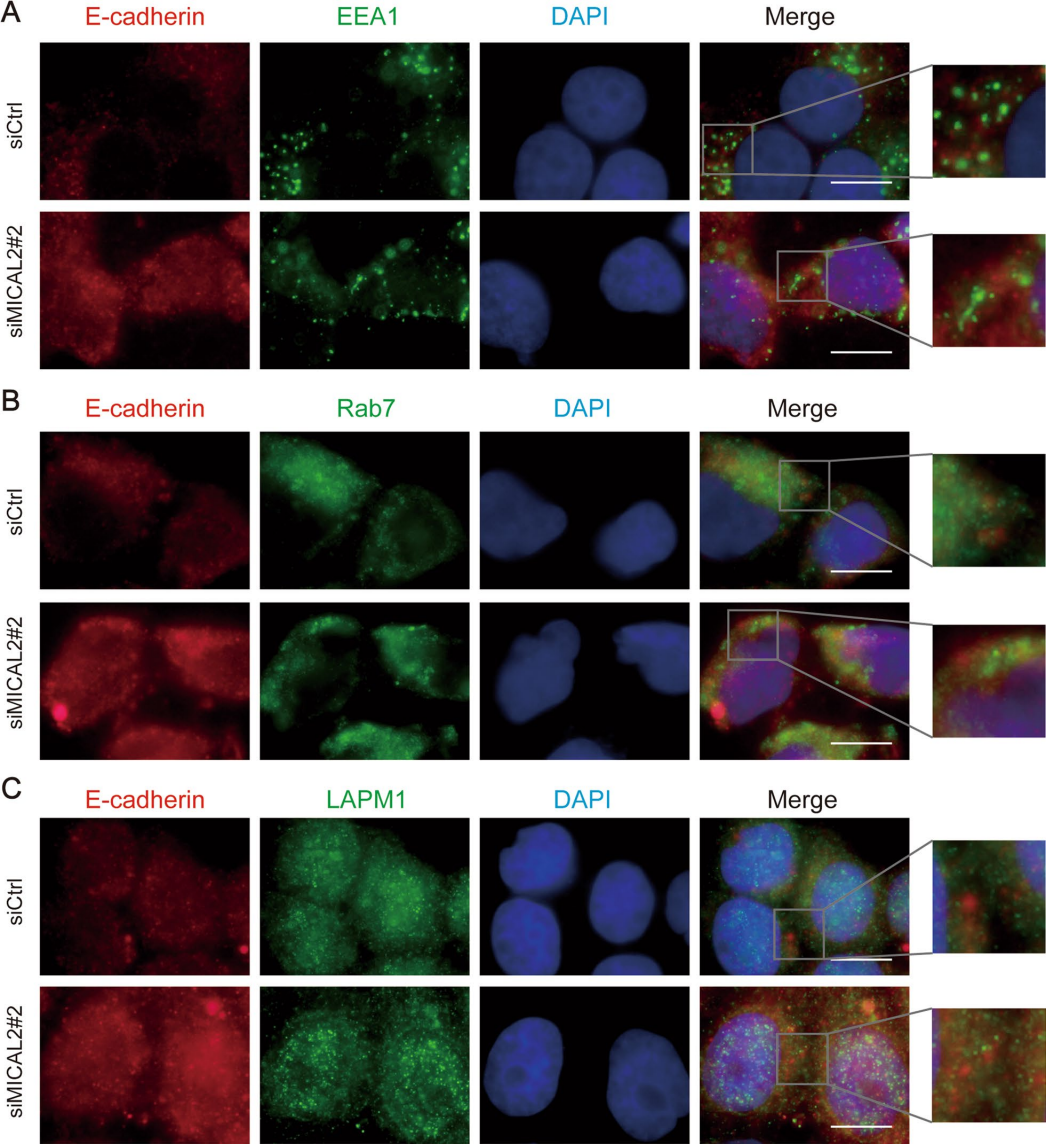
**A**-**C** The effect of MICAL2 on E-cadherin cellular localization. BGC-823 cells transfected with control siRNA or siRNA targeting MICAL2 (siMICAL2) were stained with antibodies against EEA1 (**A**), Rab7 (**B**), or LAMP1 (**C**). Endocytic markers are shown in red. E-cadherin is shown in green. Nuclei (blue) were counterstained with DAPI. Yellow indicates co-localization. Scale bar, 10 μm

To uncover the potential mechanism underlying how the silencing of MICAL2 induces E-cadherin protein expression, we evaluated E-cadherin distribution in the cytoplasm using an immunofluorescence assay. Normally, after binding to its ligands, E-cadherin undergoes dimerization and autophosphorylation. Phosphorylated E-cadherin is then ubiquitinated and enters early and late endosomes in the cytoplasm. Finally, it is degraded in lysosomes, resulting in reduced recycling of E-cadherin to the lateral membrane [26]. To further confirm the relationship between E-cadherin and MICAL2, we investigated whether E-cadherin subcellular localization was altered following MICAL2 depletion. Immunofluorescence assay results showed that relatively little E-cadherin co-localized with the early endosome marker EEA1 or the late endosomal marker Rab7, and only partially co-localized with the lysosomal marker LAMP1. Combined, these findings indicated that MICAL2 depletion did not influence E-cadherin localization in early endosomes, late endosomes, or lysosomes (Fig. 5A–C), suggesting that MCIAL2 does not modulate lysosome pathway-mediated E-cadherin degradation.

**Fig. 6:**
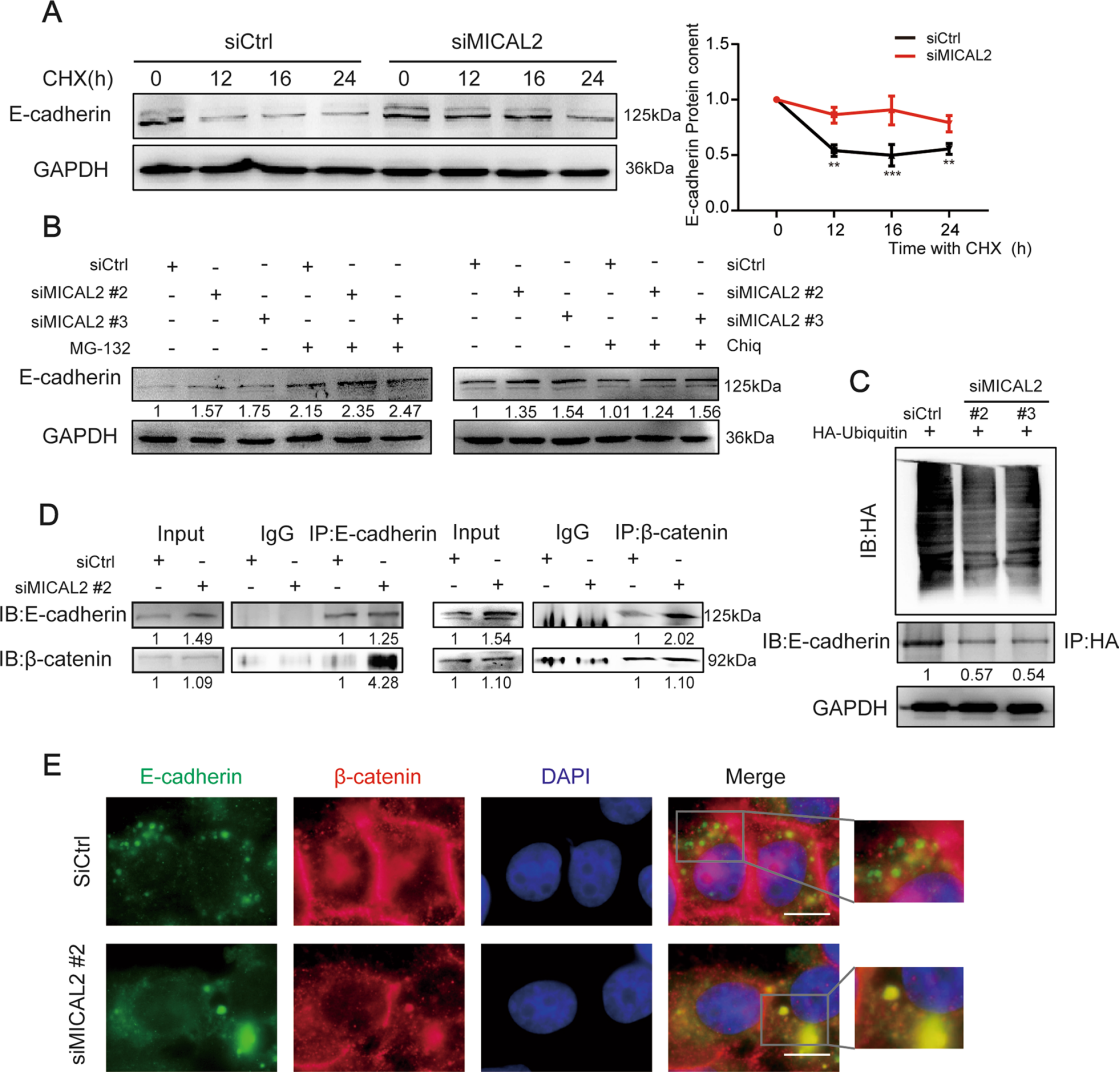
MICAL2 depletion leads to reduced E-cadherin degradation. **A** BGC-823 cells transfected with control siRNA or siRNA targeting MICAL2 (siMICAL2#2) were treated with 10 μg/mL cycloheximide (CHX, an inhibitor of protein synthesis) following which the cells were lysed and the E-cadherin protein level was determined by western blotting. GAPDH was used as the loading control. **B** BGC-823 cells transfected with siMICAL2 were treated with chloroquine (10 μM) or MG-132 (20 μM) for 24 h. Total proteins were then extracted from the lysates and subjected to western blotting for the measurement of E-cadherin expression levels. GAPDH served as the loading control. **C** BGC-823 cells were co-transfected with HA-ubiquitin and siMICAL2, following which E-cadherin ubiquitination was assayed. **D** Co-immunoprecipitation assays were used to detect the binding of endogenous E-cadherin to β-catenin in cells transfected with siMICAL2. **E** Representative immunofluorescence micrographs of BGC-823 cells stained for E-cadherin and β-catenin. Scale bar, 10 μm. **P < 0.01, ***P < 0.001

As shown in Fig. 6A, MICAL2 depletion in BGC-823 cells significantly inhibited E-cadherin degradation when CHX, a protein synthesis blocker, was added to the culture medium. To further uncover the mechanisms involved in MICAL2-mediated E-cadherin degradation, we treated cells with two inhibitors of known degradation pathways, namely, chloroquine, a lysosomal proteolysis inhibitor, and MG-132, a proteasome inhibitor. The results showed that only the proteasome inhibitor MG-132 could block MICAL2 knockdown-induced E-cadherin degradation (Fig. 6B), suggesting that MICAL2 inhibits E-cadherin degradation possibly by preventing its entry into the proteasome pathway. To further examine the mechanism underlying E-cadherin proteasomal degradation, we examined E-cadherin ubiquitylation levels in MICAL2-depleted cells. As shown in Fig. 6C, E-cadherin ubiquitylation levels were decreased in BGC-823 cells transfected with siMICAL2. The above results suggested that MICAL2 maintains E-cadherin protein levels possibly through repressing ubiquitylation-mediated E-cadherin degradation in gastric cancer cells.

### MICAL2 inhibited the formation of the β-catenin/E-cadherin complex

Wnt/β-catenin signaling has been reported to play an important role in MICAL2-induced EMT in ovarian cancer cells [22]. To uncover the potential mechanism through which MICAL2 silencing induces E-cadherin degradation, we tested β-catenin/E-cadherin complex formation in gastric cancer cells. Co-immunoprecipitation assays in BGC-823 cells confirmed that endogenous β-catenin interacted with E-cadherin (Fig. 6D) while immunofluorescence analysis also showed that β-catenin partially co-localized with E-cadherin in the same cells (Fig. 6E). Furthermore, when BGC-823 cells were treated with siMICAL2 (#2), β-catenin/E-cadherin interaction was increased, as determined by western blotting, whereas β-catenin nuclear localization was decreased, as evidenced by immunofluorescence staining results (Fig. 6E). Combined, these findings implied that MICAL2 depletion markedly inhibits E-cadherin degradation; this, in turn, promotes E-cadherin/β-catenin complex formation while also decreasing β-catenin nuclear translocation, resulting in the downregulation of Wnt/β-catenin signaling.

### The silencing of MICAL2 suppressed β-catenin nuclear localization

**Fig. 7:**
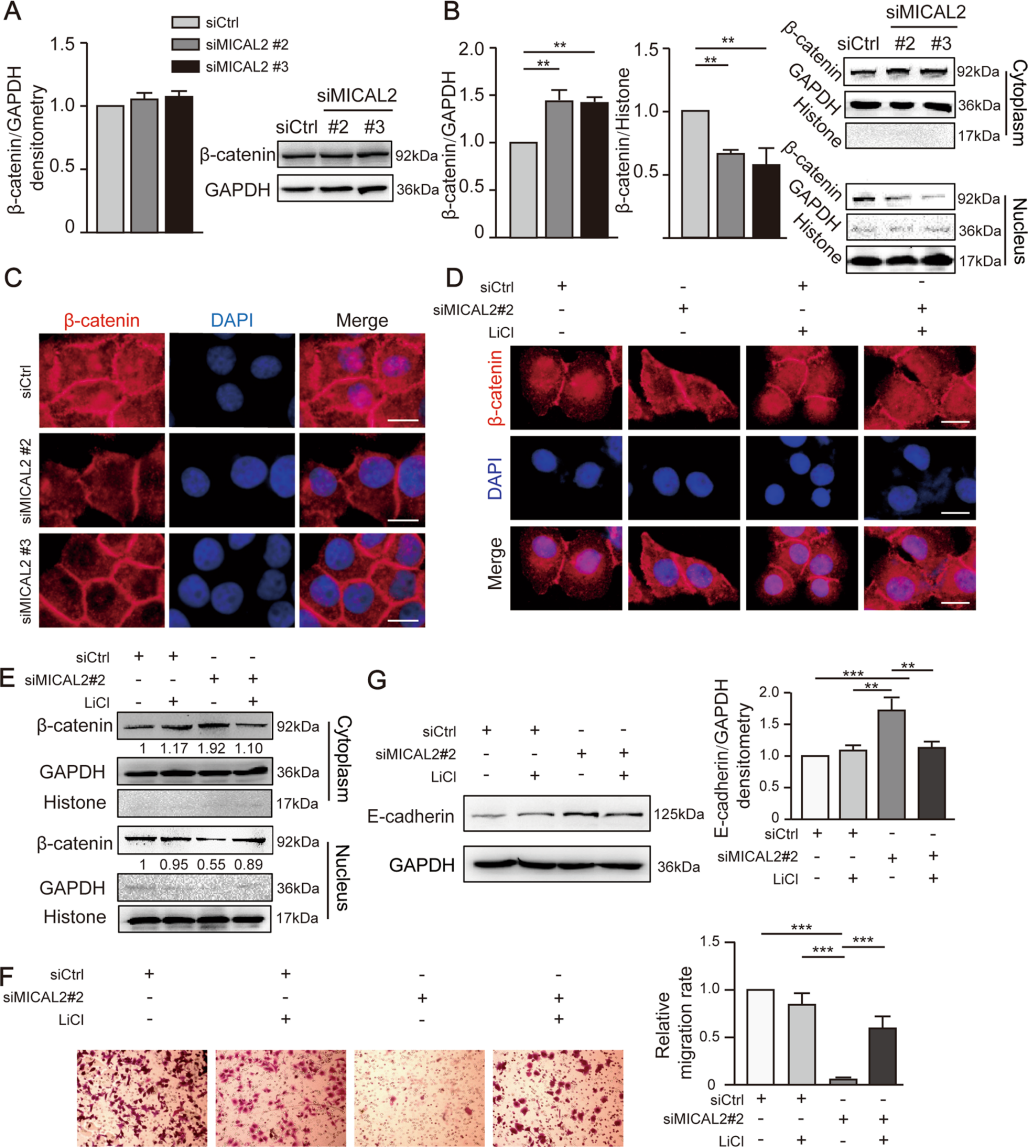
MICAL2 regulates cell migration via the Wnt/β-catenin signaling pathway. **A** BGC-823 cells were transfected with control siRNA or siRNA targeting MICAL2 (siMICAL2) for 48 h. Subsequently, total protein extracts were assessed for β-catenin levels and distribution. **B** β-catenin levels were measured in cytoplasmic and nuclear extracts obtained from BGC-823 cells transfected with siMICAL2. GAPDH served as the cytoplasmic control and histone H3 as the nuclear control. **C** Representative immunofluorescence images of β-catenin staining in MICAL2-depleted BGC-823 cells. Scale bar, 10 μm. **D** Representative immunofluorescence images of MICAL2-depleted BGC-823 cells pre-treated with LiCl. Scale bar, 10 μm. **E**–**G** MICAL2-depleted BGC-823 cells were pre-treated with LiCl, after which β-catenin protein levels in cytoplasmic and nuclear extracts (**E**), the cell migration rate (**F**), and E-cadherin levels (**G**) were determined. **P < 0.01, ***P < 0.001

β-catenin nuclear translocation can lead to the activation of the Wnt/β-catenin signaling pathway [27]. To explore the role of MICAL2 in regulating β-catenin nuclear expression, we silenced MICAL2 in BGC-823 cells using siMICAL2 #2 and #3. As shown in Fig. 7A, neither siRNA led to a significant change in total β-catenin expression levels in these cells; however, there was a significant reduction in β-catenin levels in nuclear fractions when compared with that in the cytoplasmic/membrane fractions (Fig. 7B). Immunofluorescence staining further showed that β-catenin nuclear content was decreased in MICAL2-silenced BGC-823 cells (Fig. 7C), an effect that was reversed by pre-treatment with LiCl (a GSK3β inhibitor) (Fig. 7D). The results further demonstrated that, in MICAL2-depleted BGC-823 cells, pre-treatment with LiCl increased the β-catenin protein content in nuclear extracts and the cell migration rate, reduced E-cadherin protein expression (Fig. 7E–G).

### MICAL2 regulated E-cadherin stability via activating Cdc42

**Fig. 8:**
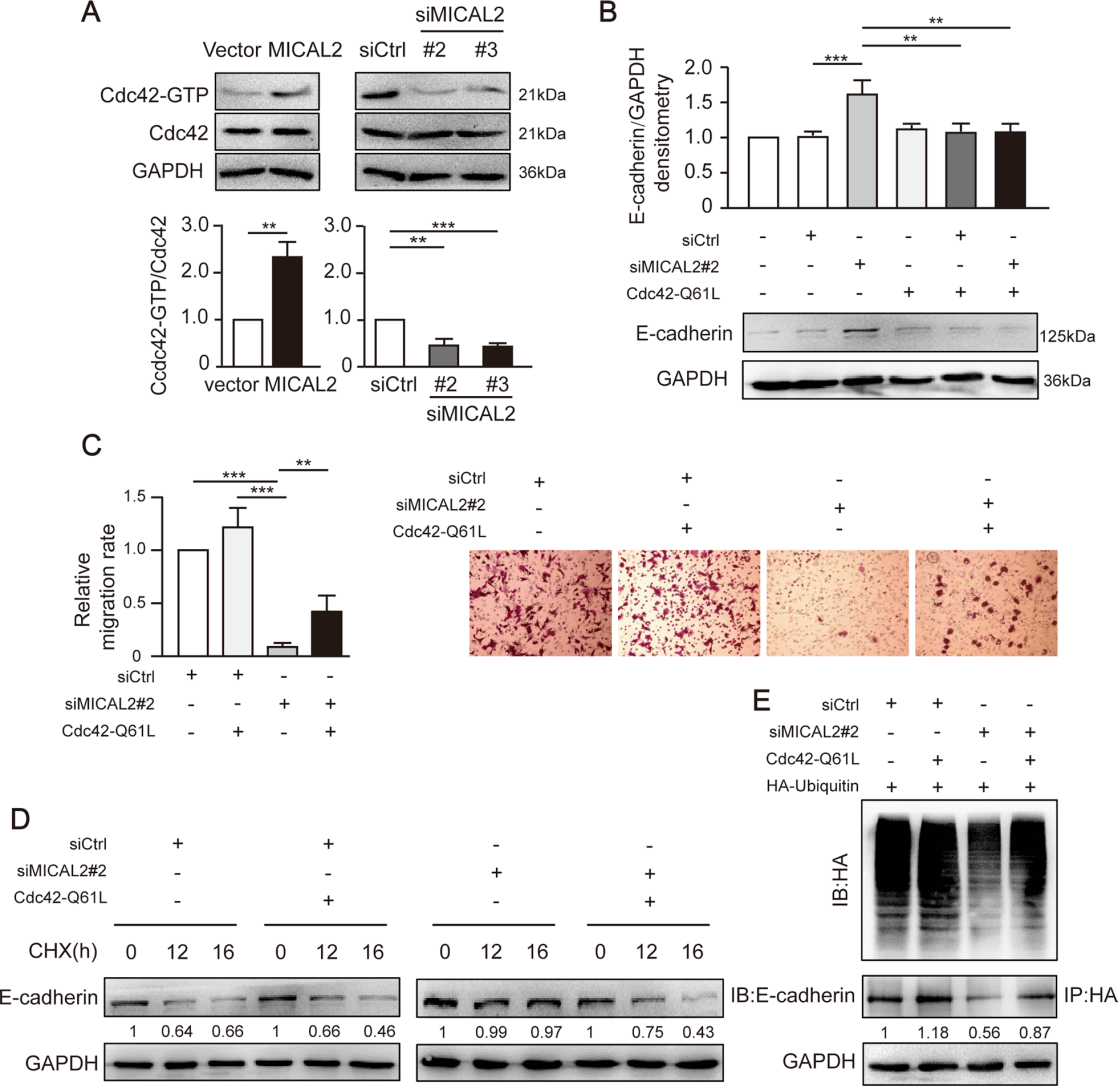
MICAL2 accelerates E-cadherin degradation via a Cdc42-dependent mechanism. **A** The activities of Cdc42 in SGC-7901 cells transfected with a MICAL2 expression plasmid and BGC-823 cells transfected with siRNA targeting MICAL2 (siMICAL2) were measured using pulldown assays. **B** MICAL2-depleted BGC-823 cells were transfected with a Cdc42-Q61L expression plasmid and total cellular proteins were extracted and assessed for E-cadherin levels by western blotting assays. **C**–**E** MICAL2-depleted cells were transfected with the Cdc42-Q61L expression plasmid and following which the cell migration rate (**C**), the E-cadherin degradation rate (**D**), and E-cadherin ubiquitination (**E**) were assayed. **P < 0.01, ***P < 0.001

**Fig. 9:**
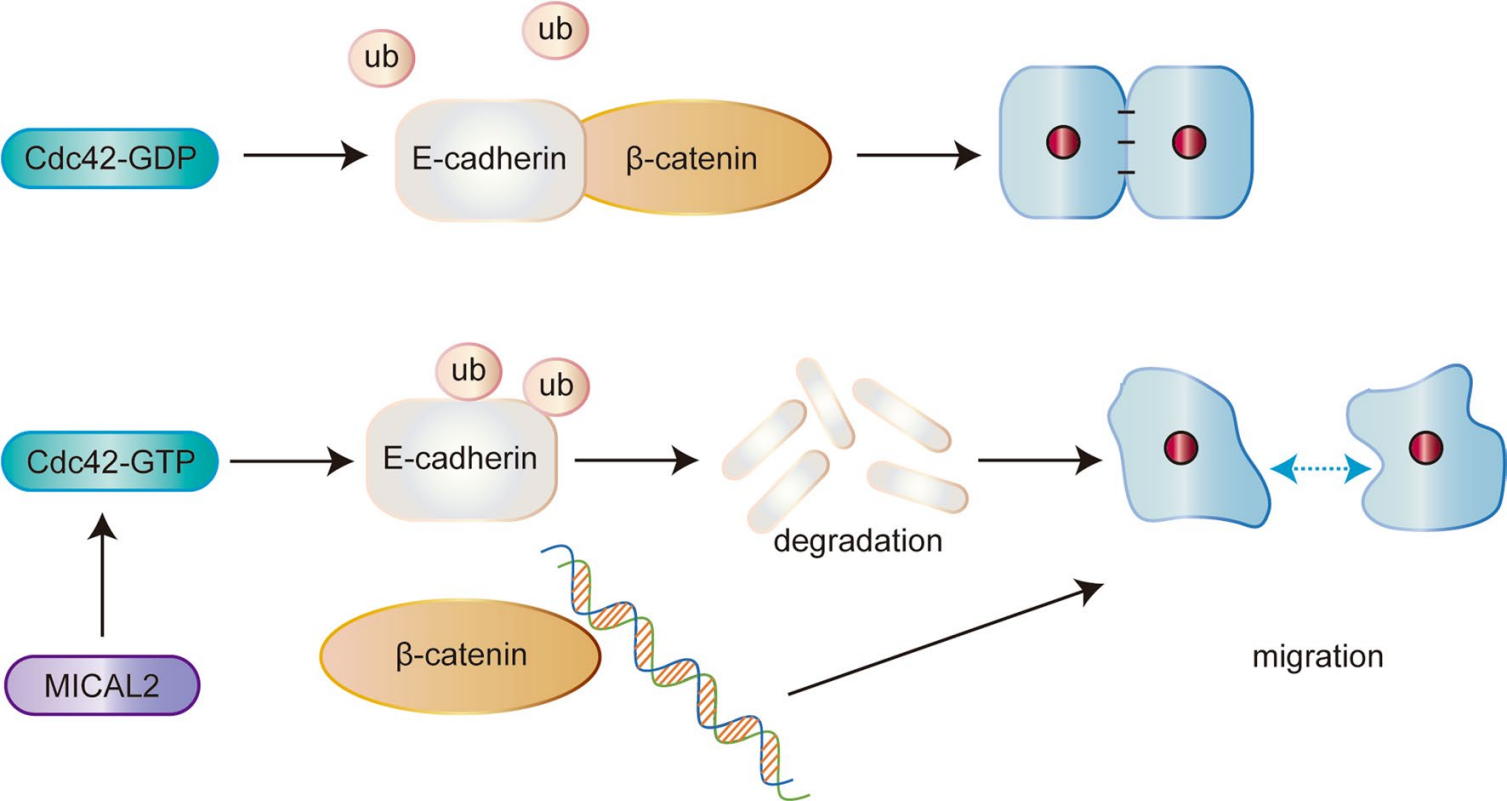
Schematic model for how MICAL2 regulates E-cadherin degradation and β-catenin-mediated cell migration. In brief, MICAL2 was identified as a novel key molecule implicated in gastric cancer cell migration. MICAL2 increased E-cadherin ubiquitination and degradation in a Cdc42-dependent manner, thereby leading to enhanced β-catenin signaling via the disruption of the E-cadherin/β-catenin complex and, consequently, the promotion of gastric cell migration

It has been reported that Cdc42 plays an important role in the internalization and degradation of receptors [28]. To further investigate the mechanism through which the silencing of MICAL2 reduces E-cadherin degradation levels, we examined Cdc42 activity by pulldown assays in BGC-823 cells transfected with siMICAL2 #2 and #3 or in SGC-7901 cells transfected with a MICAL2 expression plasmid. We found that Cdc42 activity was significantly reduced by MICAL2 knockdown and increased by MICAL2 overexpression (Fig. 8A). The transfection of Cdc42-Q61L, a constitutively active mutant of Cdc42, reversed MICAL2 silencing-induced E-cadherin protein upregulation (Fig. 8B). We further found that the cell migration rate was increased (Fig. 8C) and E-cadherin ubiquitylation and degradation were accelerated (Fig. 8D, E) when MICAL2-depleted BGC-823 cells were transfected with the plasmid expressing Cdc42-Q61L. Overall, these findings demonstrated that MICAL2 promotes E-cadherin degradation and gastric cancer cell migration in a Cdc42 activity-dependent manner (Fig. 9).

## Discussion

The results of numerous studies have demonstrated that β-catenin is a major risk factor for gastric tumor initiation, growth, metastasis, and resistance to therapy [29]. Therefore, understanding the role of β-catenin in gastric cancer cell migration as well as the underlying regulatory mechanism is important for formulating novel therapeutic strategies for the treatment of this disease. In this study, we found that MICAL2 is highly expressed in gastric cancer tissues. Moreover, we identified a novel link between MICAL2 and β-catenin in the regulation of gastric cancer cell migration, namely, that MICAL2 maintains Wnt/β-catenin signaling activation by promoting β-catenin nuclear localization, thereby enhancing the migratory ability of gastric cancer cells.

Wnt/β-catenin signaling activation is known to be associated with β-catenin nuclear translocation and negatively regulated through the degradation of β-catenin via the β-catenin destruction complex (consisting of APC, CK1, AXIN1, and GSK-3) in the cytoplasm. Here, we found that total β-catenin levels were not affected by the knockdown of MICAL2. Instead, siRNA-mediated depletion of MICAL2 led to a significant reduction in β-catenin nuclear content and a decrease in the migratory ability of gastric cancer cells, effects that were significantly attenuated by pre-treatment with LiCl, which is known to activate Wnt signaling by inhibiting GSK3β and consequently stabilizing free cytosolic β-catenin. These data suggested that MICAL2 promotes cell migration-related signaling in gastric cancer cells through regulating the cytosolic-nuclear shuttling of β-catenin. However, relatively little is known of the mechanism underlying how MICAL2 influences β-catenin translocation.

E-cadherin is a transmembrane protein expressed in almost all epithelial cells. The extracellular region of E-cadherin binds to cadherins present on adjacent cells, whereas its intracellular region contains binding sites for catenins, including β-catenin, which binds to the C-terminal of the E-cadherin cytoplasmic region. E-cadherin forms a complex with β-catenin, which, in turn, serves as an anchoring junction and acts to stabilize cell adhesion [17]. In this study, the level of E-cadherin protein was significantly increased when MICAL2 was silenced, whereas MICAL2 overexpression exerted the opposite effect. β-catenin can dissociate from the cytoplasmic tail of E-cadherin and translocate into the nucleus, thereby regulating target gene expression [30, 31]. Consequently, our observations implied that the MICAL2-mediated downregulation of E-cadherin protein levels may be the primary inducer of β-catenin nuclear translocation. This decrease in E-cadherin content was consistent with the results of Ma et al. [32], who showed that high glucose levels induced the degradation of E-cadherin in corneal epithelial cells, subsequently leading to reduced β-catenin/E-cadherin complex formation and the promotion of β-catenin nuclear translocation. That no significant changes in E-cadherin mRNA levels were observed between MICAL2-silenced and control cells suggested that MICAL2 might not regulate E-cadherin at the transcriptional level. Accordingly, we subsequently focused on the mechanisms involved in the MICAL2-induced upregulation of E-cadherin degradation.

In this study, we observed that E-cadherin protein was diffusely distributed near the cytoplasmic membrane of gastric cancer cells. This suggested that continuous E-cadherin endocytosis was already underway in these cells, thus facilitating E-cadherin degradation. The lysosomal and proteasome-based proteolytic pathways represent the two main cellular protein degradation pathways. Proteins in the MICAL family are key regulators of membrane trafficking during cell division [33]. For instance, the overexpression of MICAL-L1 was observed to lead to the accumulation of EGFR in late endosomes [34]. In the current study, we found that E-cadherin ubiquitylation levels were decreased in BGC-823 cells transfected with siMICAL2. In addition, only MG-132, a proteasome inhibitor, but not chloroquine, a lysosomal proteolysis inhibitor, could block the reduction in E-cadherin degradation resulting from MICAL2 knockdown. Our data suggested that MICAL2 promotes the ubiquitylation of E-cadherin and, consequently, the degradation of E-cadherin in the proteasome. This result supports that reported in a recent study and helps confirm the promotive effect of E-cadherin ubiquitination and degradation on the loss of hepatocyte polarity during the progress of hepatocellular carcinoma [35]. Moreover, our findings also showed that activated Wnt/β-catenin signaling further suppresses E-cadherin protein expression. Combined with the the role of MICAL2 in activating Wnt/β-catenin signaling is dependent on E-cadherin degradation, it is reasonable to think there might be an positive feedback loop for MICAL2-mediated E-cadherin low expression and Wnt/β-catenin signaling.

The most significant finding in this study was that the molecular mechanism involved in the regulation of E-cadherin degradation was largely dependent on the induction of Cdc42 activation by MICAL2. Cdc42 is known to control a wide variety of biological responses in metastatic cells, both directly and indirectly. Additionally, Cdc42 has been identified as a potential positive regulator of E-cadherin ubiquitination and degradation [28]. In the current study, we found that MICAL2 knockdown greatly suppressed Cdc42 activation, whereas MICAL2 overexpression exerted the opposite effect, indicating that MICAL2 promotes Cdc42 activation. When we analyzed the role of Cdc42 in the maintenance of E-cadherin stability in gastric cancer cells, we observed that a constitutively active form of Cdc42 significantly abrogated the upregulation of E-cadherin expression and the downregulation of cell migratory potential in MICAL2-depleted cells. Cdc42 has been reported to enhance EGFR/Src signaling by switching from an inactive GDP-bound form to an active GTP-bound one, thereby leading to E-cadherin ubiquitination [28]. Thus, it is likely that MICAL2 may accelerate E-cadherin ubiquitination and degradation in a Cdc42-dependent manner, leading to impaired E-cadherin/β-catenin binding and increased translocation of β-catenin into the nucleus. A recent study showed that MICAL2 is a key inducer of ROS production in gastric cancer cells [36]. Although ROS can reportedly suppress the activation of Cdc42GAP, a protein that is required for Cdc42-mediated GTP hydrolysis [37], the mechanism via which MICAL2 regulates Cdc42 activation, as shown in this study, remains to be elucidated.

In conclusion, we identified a novel role for MICAL2 in the regulation of gastric cancer cell migration, and showed that the underlying mechanism involved the impaired binding of E-cadherin to β-catenin in the cytoplasm, subsequently leading to the accumulation of β-catenin in the nucleus. We further showed that this effect of MICAL2 was likely mediated via the activation of Cdc42, which led to increased E-cadherin ubiquitination and degradation. To the best of our knowledge, this was the first study to report the degradation of E-cadherin mediated by MICAL2. Further advances in our understanding of the function of MICAL2 will likely have important implications for the treatment of gastric cancer.

## Data Availability

The datasets supporting the conclusions of this article are included within the article.

## Abbreviations

CHX: Cycloheximide
Chlq: Chloroquine diphosphate
Co-IP: Co-immunoprecipitation
EMT: Epithelial-to-mesenchymal transition
GTEx: Genotype tissue expression
MICALs: The molecules interacting with CasL
STAD: Stomach adenocarcinoma
TCGA: The cancer genome atlas
